# Adaptive Resistance of *Staphylococcus aureus* to Cefquinome Sulfate in an In Vitro Pharmacokinetic Model with Transcriptomic Insights

**DOI:** 10.3390/microorganisms13020329

**Published:** 2025-02-02

**Authors:** Yue Hu, Hao Zhu, Xingbo Zhang, Yuhui Wu, Jingtao Li, Nan Li, Zhanbo Cai, Yuhui Yang

**Affiliations:** College of Tropical Agriculture and Forestry, Hainan University, Haikou 570228, China; 15289852251@163.com (Y.H.); hy0219028@muhn.edu.cn (H.Z.); 15105690350@163.com (X.Z.); 18417266830@163.com (Y.W.); 15839127638@163.com (J.L.); 13361319390@163.com (N.L.); 17762524946@163.com (Z.C.)

**Keywords:** cefquinome sulfate, *Staphylococcus aureus*, pharmacokinetic model in vitro, adaptive drug resistance, transcriptional sequencing

## Abstract

Cefquinome sulfate has a strong killing effect against *Staphylococcus aureus* (*S. aureus*), but bacterial resistance has become increasingly widespread. Experiments were conducted to investigate the pattern of adaptive resistance of *S. aureus* to cefquinome sulfate under different dosage regimens by using pharmacokinetic-pharmacodynamics (PK-PD) modeling, and the adaptive-resistant bacteria in different states were screened and subjected to transcriptomic sequencing. The results showed that the minimum inhibitory concentration of Staphylococcus aureus under the action of cefquinome sulfate was 0.5 μg/mL, the anti-mutation concentration was 1.6 μg/mL, and the mutation selection window range was 0.5~1.6 μg/mL. In the in vitro pharmacokinetic model to simulate different dosing regimens in the animal body, there are certain rules for the emergence of adaptive drug-resistant bacteria: the intensity of bacterial resistance gradually increased with culture time, and the order of emergence was tolerant bacteria (TO) followed by persistent bacteria (PE) and finally resistant bacteria (RE). The sequence reflected the evolution of adaptive drug resistance. Transcriptome Gene Ontology (GO) analysis revealed that differentially expressed genes were involved in cellular respiration, energy derivation by oxidation of organic compounds, and oxidation–reduction processes. The differentially expressed genes identified functioned in the synthesis of cell membranes, cytoplasm, and intracellular parts. A Kyoto Encyclopedia of Genes and Genomes (KEGG) pathway analysis found that 65 genes were differentially expressed after cefquinome sulfate treatment, of which 35 genes were significantly upregulated and 30 genes were significantly downregulated. Five genes, sdhB, sdhA, pdhA, lpdA, and sucC, may be involved in network regulation. This study revealed the cross-regulation of multiple metabolic pathway networks and the targets of network regulation of *S. aureus* to produce adaptive drug resistance. The results will provide guidance for clinical drug use in animals infected with *S. aureus*.

## 1. Introduction

*Staphylococcus aureus* was discovered by the surgeon Alexander Ogston from the abscess of a patient’s ulcer. *S aureus* is a Gram-positive bacterium and is a common foodborne pathogen distributed in nature [1]. The bacteria cause a variety of infections and diseases in humans and animals. These include skin and soft tissue infections [2], pneumonia [3], endocarditis [4], and mammary gland infections [5] in domestic animals [6]. *Staphylococcus aureus* is considered to be one of the major infectious pathogens causing 5–15% of dairy cow mastitis cases [7]. Intramammary infection of *S. aureus* can cause huge losses to the dairy industry; affect the health, welfare, and productivity of dairy cows; and increase the risk of disease transmission within the herd or premature elimination of diseased animals [8]. Since the first methicillin-resistant *S. aureus* (MASR) strain was discovered, the drug resistance of *S. aureus* has gradually increased, and resistant strains have become widespread; at present, drug resistance is the main reason for the failure of treatments for bacterial diseases.

Drug resistance can be divided into inherent resistance, acquired resistance, and adaptive resistance [9]. Adaptive drug resistance refers to the rapid response of bacteria under non-lethal environmental pressure and is the temporary enhancement of drug resistance generated by bacteria through gene regulation and protein expression [10]. This is the link between inherent drug resistance and acquired drug resistance and the main reason for the treatment failure of many clinical bacterial infections [11,12,13]. Adaptive resistance results from the interaction of multiple bacterial drug resistance mechanisms [14]. Previous studies have shown that these mechanisms include the binary regulatory system of bacteria [15], the SOS response [16], and the adaptive responses of bacteria to environmental changes. The latter include epigenetic factors [17,18], effector pump and porin expression [19], persistent bacteria [20,21], biofilm formation [22,23], and increased mutation rates [24]. After receiving environmental signals, bacteria can immediately react to factors that threaten their survival, and thus, their resistance is gradually increased. Therefore, bacterial adaptive drug resistance is the result of the combined action of multiple pathways, and there are correlations among these pathways [24,25].

Cefquinome sulfate is a β-lactam antibiotic and the only fourth-generation cephalosporin antibiotic used to treat animals [26]. The antibiotic has excellent pharmacokinetic characteristics, a wide antibacterial spectrum, strong antibacterial activity, and high bioavailability [27]. Cefquinome sulfate is suitable for parenteral administration and is widely used in disease prevention and treatment of poultry and livestock, primarily for the treatment of cow mastitis, digestive tract and respiratory tract diseases of pigs [28], chicken dysentery, infectious serositis of ducks [29], dog pyoderma, and other bacterial diseases. Cefquinome sulfate has bactericidal effects against *S. aureus*, *Streptococcus*, *Pseudomonas aeruginosa*, and Enterobacteriaceae, as well as MASR. With the application of cefquinome sulfate, bacterial resistance to it has gradually emerged. It is generally believed that the mechanism of drug resistance of *Staphylococcus aureus* to cefquinome sulfate is the production of an active binding site with low affinity for β-lactam antibiotics—PBP2a, which can replace the PBPS family of penicillin-binding proteins originally bound with drugs—and promotes the synthesis of bacterial cell walls without being specifically bound [30].

The emergence of antibacterial drugs has brought enormous benefits to human health and well-being. However, due to the abuse of antibiotics in farming, bacterial resistance to cefquinome sulfate has emerged. Studies have found that *S. aureus* can produce rapid and regular adaptive resistance to cefquinome sulfate [31]. Therefore, to explore the network regulatory targets for the adaptive resistance of *S. aureus* to cefquinome sulfate and provide empirical support for the control of adaptive resistance, this study used PK/PD modeling to induce adaptive resistance in bacteria with different drug resistance states and strengths. We performed transcriptomic sequencing on these resistant bacteria. Moreover, bioinformatics was used to analyze the results to characterize the network regulation and gene expression underlying adaptive resistance.

## 2. Materials and Methods

### 2.1. Bacterial Strains, Reagents, and Growth Conditions

*Staphylococcus aureus* (ATCC6538) was purchased from the China Institute of Veterinary Drug Control (Beijing, China). Cefquinome sulfate (68.6% purity, Lot 9017208H) was purchased from QILU Synva Pharmaceutical Co., Ltd. (Dezhou, China). Mueller–Hinton (MH) Broth and Mueller–Hinton (MH) AGAR were purchased from Qingdao Hi-Tech Industrial Park Hope-Technology Co., Ltd. (Qingdao, China). *Staphylococcus aureus* was grown in MH broth in a 37 °C incubator.

### 2.2. Formulation of Antibacterial Drugs

Cefquinome sulfate (75 mg; purity 68.6%) was dissolved in a 10 mL volumetric bottle to obtain a 5.120 mg/mL stock solution. The solution was stored in a −80 °C refrigerator away from light for later use. Nutrient broth medium and nutrient AGAR solid medium were prepared according to the medium instructions.

### 2.3. Sensitivity Test In Vitro

The minimum inhibitory concentration (MIC) was measured by the micro broth dilution method according to the Standards of Clinical and Laboratory Standards Institute (CLSI). The mutation preventive concentration (MPC) of cefquinome sulfate against *S. aureus* was determined by the AGAR double dilution method. The concentration range between MIC and MPC is defined as the mutation-selection window (MSW) of cefquinome sulfate against *S. aureus*.

### 2.4. Establishment of an In Vitro PK/PD Model for Cefquinome Sulfate

A PK/PD model for cefquinome sulfate was established according to the test method of Linglin Gao [31]. A special 500 mL double-layer beaker was used as the only room in the one-room model, 300 mL of sterile MH broth was loaded into it, and a rubber plug was inserted. The beaker was placed on a magnetic stirrer and continuously stirred at a frequency of 5 HZ, and the temperature of the broth in the beaker was maintained at 37 °C through a constant temperature circulating water bath. The peristaltic pump is used to pump the broth in and out at the same rate to decrease the drug concentration. A single colony was selected from the bacterial solid medium and inoculated into 10 mL sterile MH broth and then placed on a constant temperature shaking table and incubated at 37 °C for 18 h. After shaking, the bacterial solution in the logarithmic phase was taken out, and then, 100 μL of the bacterial solution was inoculated in a double-layer beaker. The drug was administered after 12 h of bacterial growth. The administration regimen was as follows: the elimination half-life was 2.5 h; the concentrations of cefquinome sulfate were 2 μg/mL/12 h (n = 3), 3 μg/mL/12 h (n = 3), and 5 μg/mL/12 h (n = 3). The runtime of the model was 72 h. In the model, 1.5 mL of broth was collected at intervals of 6 h after administration.

### 2.5. Determination of the Peristaltic Pump Flow Rate and Frequency

The elimination half-life (t_1/2_) was set to 2.5 h. The flow rate of the peristaltic pump was determined according to the elimination half-life. The calculation formula for the half-life was R = K × V; K = ln 2/t_1/2_ ≈ 0.693/t_1/2_, where K is the elimination rate and V is the volume of the central chamber. The peristaltic pump operated at six different gradient frequencies, and the pumped broth was weighed three times within 10 min. The average flow rate was calculated, and the linear regression equation was obtained to determine the peristaltic pump frequency.

### 2.6. Determination of MIC, MDK_99_, MDK_99.99_, and Drug Sensitivity

The MIC of the bacterial solution at each time point was determined according to the micro broth dilution method. After 24 h, the reading result was compared to the MIC of a bacterial standard at 0 h. For those strains that had values greater than the MIC of the bacterial standard, the relevant sensitivity of recovery was determined. For strains having values less than or equal to the MIC of standard bacteria, the minimum time required to kill 99% of bacteria (MDK_99_) and the minimum time required to kill 99.99% of bacteria (MDK_99.99_) were measured.

### 2.7. Transcriptome Sequencing

Three different states of adaptive drug-resistant bacteria were screened from the above 3 μg/mL/12 h dose model test; in order, these were tolerant bacteria (TO), persistent bacteria (PE), and resistant bacteria (RE). Three adaptive drug-resistant bacteria and the standard original strain (OR) were sent to Wuhan SeqHealth Tech Co., Ltd. (Wuhan, China) for transcriptome sequencing. The four groups were labeled TO, PE, RE, and OR, and three biological replicates were prepared for each group.

### 2.8. Data Processing

SPSS 21.0 software was used for statistical analysis of the test data. Paired *t*-tests were performed on the MSW results of cefquinome sulfate, and one-way analysis of variance (ANOVA) was used for other data. The PK/PD model experimental results were expressed as mean ± standard error. *p* < 0.05 was used as the criterion for the significance of differences.

## 3. Results

### 3.1. Sensitivity Test Results In Vitro

The results of the in vitro sensitivity test are shown in Table 1. The MIC, MPC, and MSW values of *S. aureus* for cefquinome sulfate were 0.5, 1.6, and 0.5–1.6 μg/mL, respectively.

### 3.2. Model Flow Rate and Peristaltic Pump Frequency

**Figure 1:**
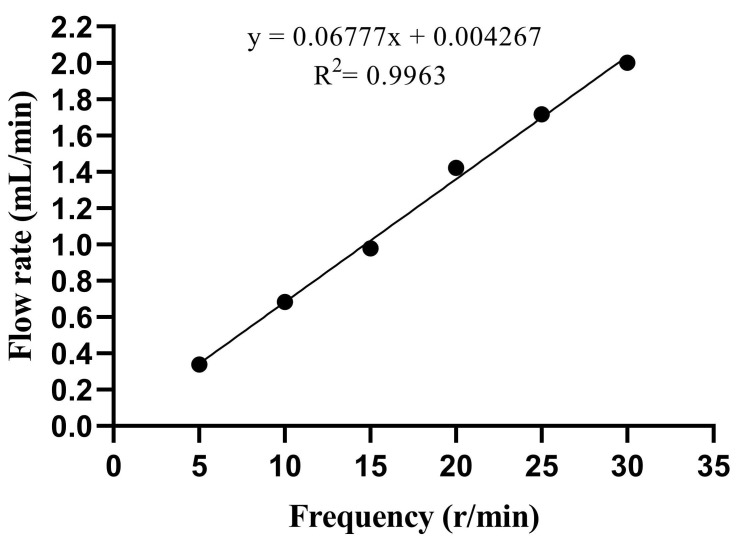
Linear analysis of flow rate. x represents the frequency (r/min), and y represents the flow rate (mL/min).

The elimination half-life was estimated as t = 2.5 h, and the flow calculation yielded a value of R = 1.386 mL/min. Figure 1 shows the standard curve for the peristaltic pump in the range of 5–30 r/min. The linear equation is *y* = 0.06777*x* + 0.004267, R^2^ = 0.9963, and the linear relationship was significant. The *y*-axis represents the flow rate (mL/min), and the *x*-axis represents the frequency (r/min). According to the linear equation, when t = 2.5 h, the corresponding frequency is 20 r/min.

### 3.3. Screening Drug-Resistant Bacteria in Different States

**Figure 2:**
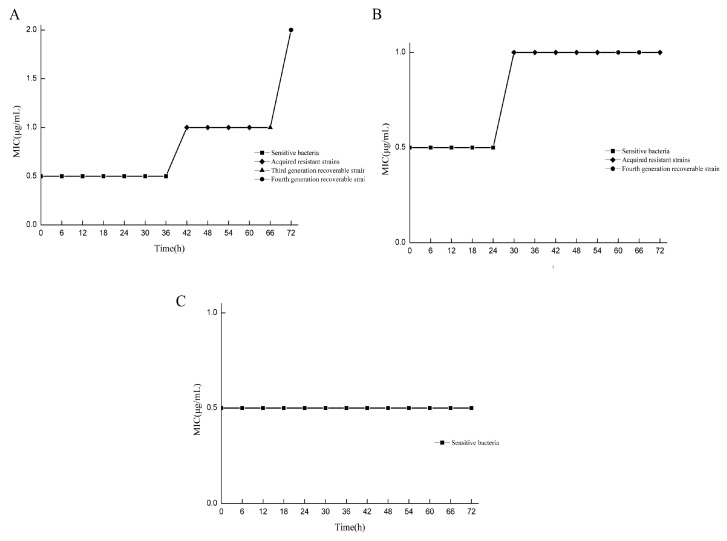
Screening drug-resistant bacteria in different states. MIC comparison results between resistant bacteria and standard strains at different doses at different times. (**A**) Under the condition of the 2 μg/mL/12 h dose model, the drug resistance intensity of resistant bacteria at 66 h and 72 h were 2MIC and 4MIC, respectively; (**B**) under the condition of the 3 μg/mL/12 h dose model, the resistance intensity of drug-resistant bacteria at 60 h and 66 h were 2MIC and 2MIC, respectively; (**C**) the 5 μg/mL/12 h dose model condition.

The MIC values of drug-resistant bacteria in various states at different doses and at different times were compared with those of standard strains. All strains were assigned to two categories, and the induced strains were preliminarily classified. Under the 2 μg/mL/12 h dose model, drug-resistant bacteria with MIC values equal to those of the standard strains were screened at 6–36 h, while drug-resistant bacteria with MIC values greater than those of the standard strains were screened at 42–72 h, and only the drug-resistant bacteria at 66 h and 72 h could recover their drug sensitivity (Figure 2A). In the 3 μg/mL/12 h dose model, drug-resistant bacteria with MIC values equal to those of the standard strains were screened from 6 to 24 h, and drug-resistant bacteria with MIC values greater than those of the standard strains were screened from 30 to 72 h. Only the drug-resistant bacteria at 60 h and 66 h could recover their sensitivity to the drug (Figure 2B). For the 5 μg/mL/12 h dose model, all the MIC values of the bacteria were equal to those of the standard strains (Figure 2C). In the determination of sensitivity recovery, the strains with unrecoverable resistance through successive generations were named as “acquired resistant strains”. For the strains whose resistance could be recovered through continuous passage, the intensity of resistance was determined, and they were referred to as “adaptive resistant strains with different resistance strengths”. Under the condition of the 2 μg/mL/12 h dose model, the strains obtained at 66 h and 72 h were the 2MIC and 4MIC adaptive resistant strains, respectively. Under the condition of the 3 μg/mL/12 h dose model, the strains obtained at 60 h and 66 h were the 2MIC and 2MIC adaptive resistant strains, respectively.

### 3.4. Determination of MDK_99_ and MDK_99.99_

**Figure 3:**
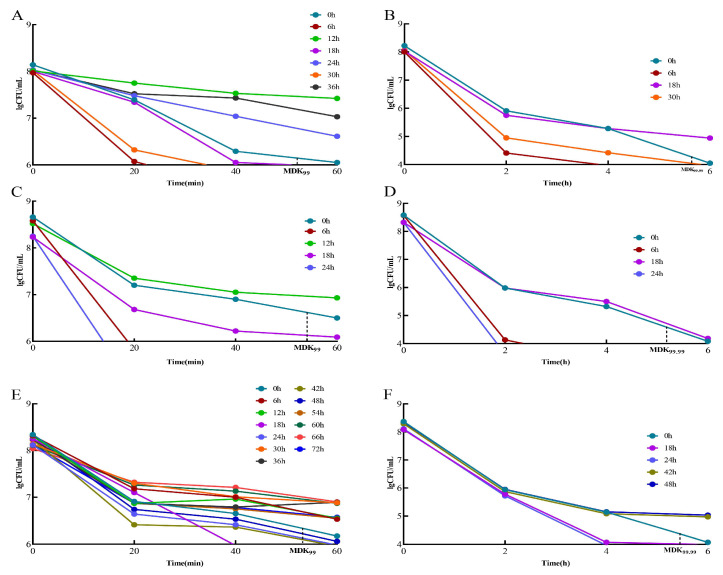
Screening of tolerant adaptive resistant bacteria and persistent adaptive resistant bacteria. Comparison of MDK99 and MDK99.99 of resistant bacteria with standard strains at different doses at different times. (**A**) Comparison of 2 μg/mL/12 h dose model bacteria MDK99; (**B**) comparison of model bacteria MDK99.99 at 2 μg/mL/12 h dose. (**C**) Comparison of 3 μg/mL/12 h dose model bacteria MDK99; (**D**) comparison of model bacteria MDK99.99 at 3 μg/mL/12 h dose; (**E**) comparison of 5 μg/mL/12 h dose model bacteria MDK99; (**F**) comparison of model bacteria MDK99.99 at 5 μg/mL/12 h dose.

When the MDK_99_ value of a strain was greater than that of the standard strain, it was considered a “tolerant” adaptive drug-resistant bacterial strain. The strains with values greater than the standard strain MDK_99.99_ were considered “persistent” adaptive drug-resistant bacteria. Strains with MDK_99.99_ values lower than that of the standard strain are referred to as “drug-sensitive strains”. In the 2 μg/mL/12 h dose model, the “tolerant” adaptive drug-resistant bacteria were screened initially and were obtained at 12, 24, and 36 h (Figure 3A). The “persistent” adaptive drug-resistant bacteria were screened at 18 h (Figure 3B). In the 3 μg/mL/12 h dose model, the “tolerant” adaptive drug-resistant bacteria were initially obtained at 12 h (Figure 3C), followed by “persistent” adaptive drug-resistant bacteria at 18 h (Figure 3D). In the 5 μg/mL/12 h dose model, “tolerant” adaptive drug-resistant bacteria were first screened at 6, 12, 30, 36, 54, 60, 66, and 72 h (Figure 3E), and “persistent” adaptive drug-resistant bacteria were screened at 42 h and 48 h (Figure 3F).

### 3.5. Bacterial RNA

The extracted bacterial RNA was tested for completeness using a Qsep 100 and for purity using a Nanodrop spectrophotometer. The RNA content of each group was greater than 1 μg, the A260/280 values of the samples were greater than 1.5, and the RQN value of the samples was greater than 4. The requirements for the RNA content, purity, and integrity of each group of samples met the experimental requirements, and thus, subsequent experiments could be conducted (Table S1).

### 3.6. Differential Gene Expression

**Figure 4:**
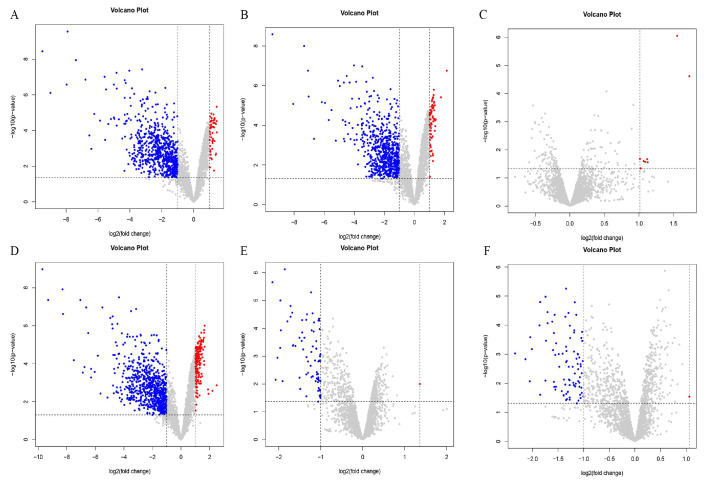
Volcano plot among four groups of strains. Horizontal coordinate log2(fold change): logarithm of differential folds with a base of 2; vertical coordinate -log10(*p*-value): negative logarithm of *p* value with a base of 10; gray dots represent genes that were not differentially expressed, blue dots represent genes that were differentially downregulated, and red dots represent genes that were differentially upregulated. (**A**) PEvsOR group differential gene volcano map. (**B**) REvsOR group differential gene volcano map. (**C**) REvsPE group differential gene volcano map. (**D**) TOvsOR group differential gene volcano map. (**E**) TOvsPE group differential gene volcano map. (**F**) TOvsRE group differential gene volcano map.

Differentially expressed genes were identified using the criteria of an absolute value of log FC > 1 and a *p*-value < 0.05. The results are shown in Figure 4. There were 58 upregulated genes and 705 downregulated genes in the PE and OR groups (Figure 4A). There were 67 differentially upregulated genes between the RE and OR groups and 665 differentially downregulated genes (Figure 4B). There were eight differentially upregulated genes and no differentially downregulated genes between the RE and PE groups (Figure 4C). There were 176 differentially upregulated genes and 764 differentially downregulated genes between the TO and OR groups (Figure 4D). Between the TO group and the PE group, there was one differentially upregulated gene and 74 differentially downregulated genes (Figure 4E). Between the TO group and the RE group, there was one differentially upregulated gene and 87 differentially downregulated genes (Figure 4F). There were significant differences between the three experimental groups and the original group.

### 3.7. Cluster Analysis of Differentially Expressed Genes

**Figure 5:**
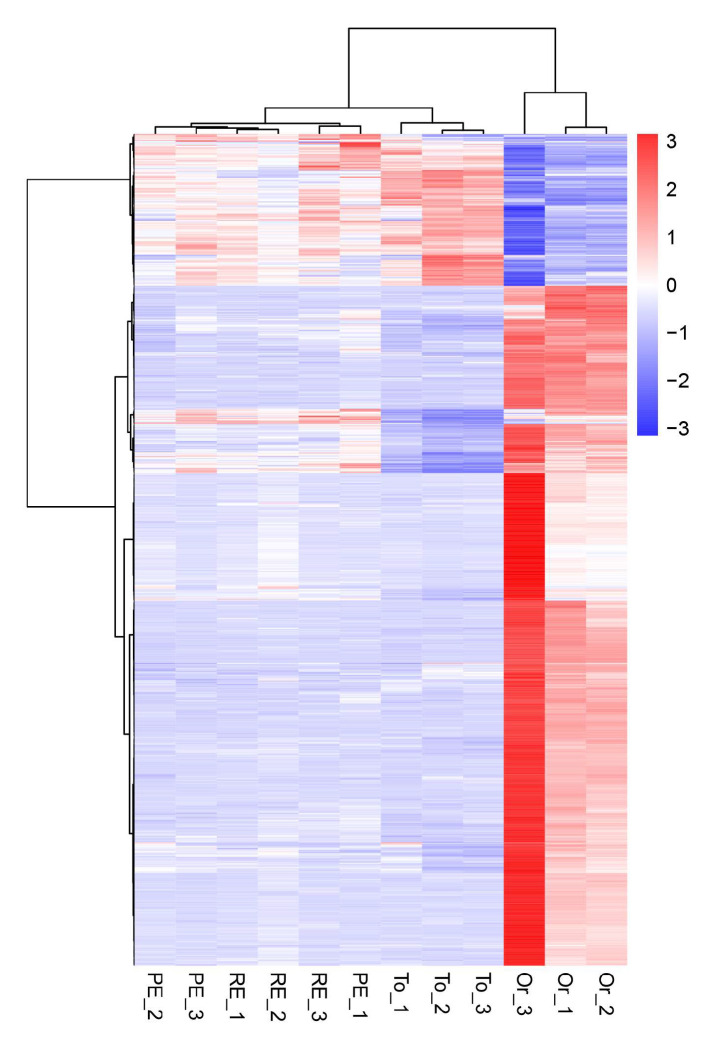
Overall hierarchical clustering of all differentially expressed genes in all comparison groups. Red indicates highly expressed genes, and blue indicates low-expressed genes. The *x* axis shows the different samples, and the *y* axis shows the gene names.

In the differential gene cluster diagram for the three adaptive drug-resistant strains and the original strain (Figure 5), the patterns of gene expression regulation of the strains in the same groups were similar, while the pattern of gene expression in the three adaptive drug-resistant strains and the original strain was significantly different.

### 3.8. GO Gene Ontology Analysis

**Figure 6:**
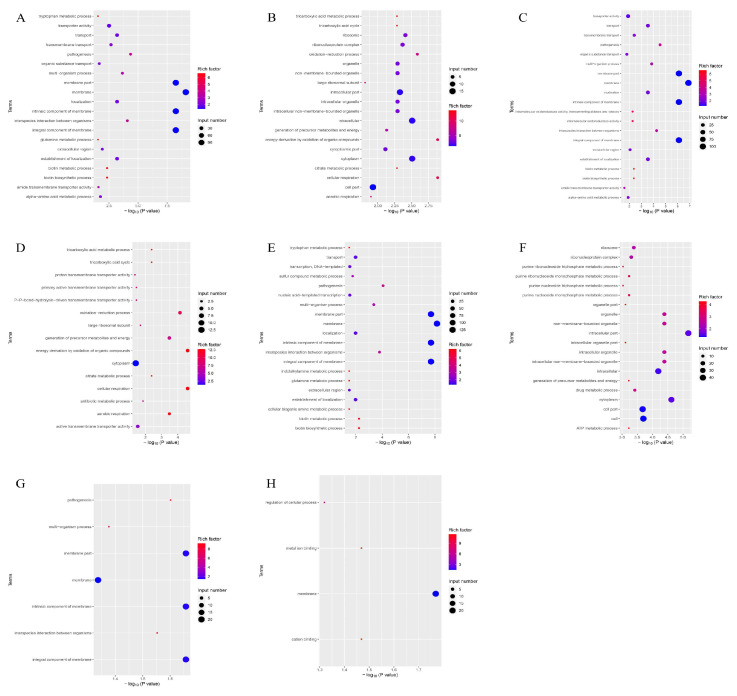
Enrichment map of GO functional annotation of differential genes of the four groups of strains. The horizontal coordinate is the significance of the enrichment (expressed as -log10 (*p* value); the larger the value, the more significant the enrichment), and the vertical coordinate is the pathway name. (**A**) GO-enriched bubble map of downregulated genes in the PEvsOR group. (**B**) GO-enriched bubble map of upregulated genes in the PEvsOR group. (**C**) GO-enriched bubble map of downregulated genes in the REvsOR group. (**D**) GO-enriched bubble map of upregulated genes in the REvsOR group. (**E**) GO-enriched bubble map of downregulated genes in the TOvsOR group. (**F**) GO-enriched bubble map of upregulated genes in the TOvsOR group. (**G**) GO-enriched bubble plot of downregulated genes in the TOvsPE group. (**H**) GO-enriched bubble plot of downregulated genes in the TOvsRE group.

According to the GO function analysis of the differentially expressed genes of the four groups (Figure 6), the genes participated in cellular respiration, energy derivation by oxidation of organic compounds, and oxidation–reduction processes. These genes control the synthesis of membranes, cytoplasm, and intracellular parts.

### 3.9. KEGG Pathway Analysis

**Figure 7:**
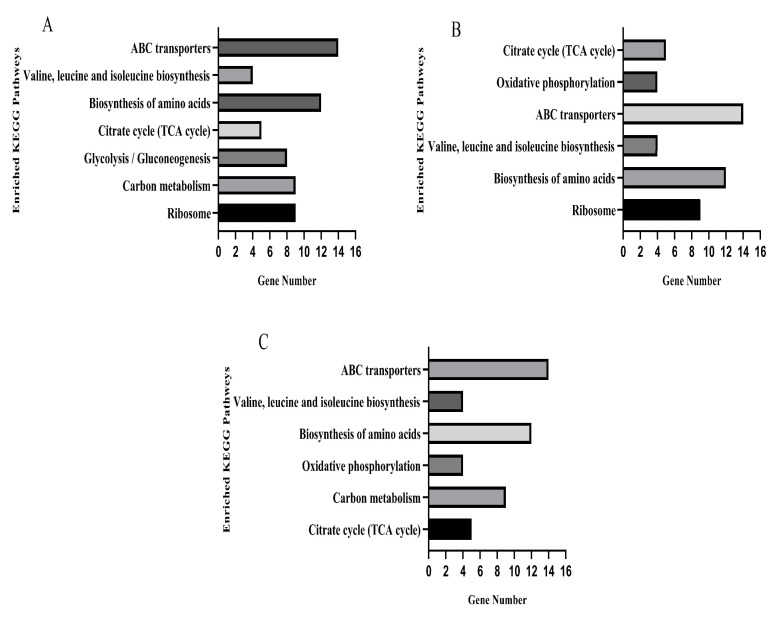
KEGG pathways significantly enriched for differential genes in three sets of strain samples versus the original standard strain. (**A**) TOvsOR differential genes were significantly enriched in the KEGG pathway. (**B**) PEvsOR differential genes were significantly enriched in the KEGG pathway. (**C**) REvsOR differential genes were significantly enriched in the KEGG pathway.

The KEGG database was used for pathway enrichment analysis for differentially expressed genes. After KO annotation of the genes, there were seven significantly enriched pathways in the TO and OR groups (Figure 7A), six significantly enriched pathways in the PE and OR groups (Figure 7B), and six significantly enriched pathways in the RE and OR groups (Figure 7C). The gene expression pathways of the three groups of drug-resistant bacteria with different states were involved in the biosynthesis of amino acids, ABC transporters, valine, leucine, and isoleucine and in the citrate cycle (TCA cycle).

There were 65 differentially expressed genes in the upregulated and downregulated KEGG pathways in the samples of the three groups compared to the original standard strains, of which 35 were significantly upregulated, and 30 were significantly downregulated. Five genes in the citrate cycle pathway, sdhB, sdhA, pdhA, lpdA, and sucC, were upregulated (Table S2).

## 4. Discussion

It is evident that drug resistance has increased due to the extensive use of antibiotics [32]. Pharmacokinetics-pharmacodynamics (PK-PD) models are important for the analysis of drug resistance. PK/PD models can simulate the dynamic changes of drugs in vivo through in vitro experiments, and then, a comprehensive analysis of the relevant drug parameters can be conducted [33]. The in vitro model can greatly reduce the use of experimental animals, can avoid the errors caused by individual differences of animals, and are convenient for human experiments with strong controllability. This study considered bacterial strains with the same elimination half-life, the same administration interval, and different administration concentrations. In the 2 μg/mL/12 h and 3 μg/mL/12 h groups, the intensity of drug resistance increased with time, while in the 5 μg/mL/12 h group, when the concentration exceeded a certain MPC threshold, the drug resistance intensity of bacteria increased with time. The occurrence of acquired drug resistance in bacteria could be eliminated, and there was no generation or enrichment of acquired drug resistance, consistent with the research results of Linglin Gao et al. [31].

The gene expression pathways of the three groups of drug-resistant bacteria in different states were involved in the biosynthesis of amino acids, ABC transporters, valine, leucine, and isoleucine and in the citrate cycle (TCA cycle). These results indicate that the same pathways are required for the generation of various adaptive drug-resistant bacteria, suggesting that these four pathways may be the most basic for the resistance mechanism of *S. aureus*. The expression levels of five genes in the citrate cycle were upregulated, suggesting that these five genes may be the key to the regulation of an adaptive resistance network.

As one of the largest and oldest membrane protein families, ABC transporters exist widely in various organisms. As early as the 1970s, this class of transporters was identified in studies of nutrient absorption by bacteria [34]. Subsequently, multidrug resistance (MDR) was shown to involve ABC transporters due to their ability to expel foreign substances from the cell in a way that reverses the concentration gradient. MDR has been further studied by researchers in the field of clinical treatment [35], and the process requires the energy of ATP [36,37]. The transport function of the ABC transporter family is divided into two transport modes: outward and inward [38,39]. The outward transporter functions in detoxification and can expel substances that are not conducive to cell growth such as antibiotics and fatty acids. ABC transporters are involved in antibiotic resistance of bacteria [40], and some bacterial ABC transporters can transfer antibiotics from the external environment to the bacterial cells, thereby reducing the killing effect of antibiotics. Relevant studies have shown that the multidrug efflux pump of ABC transporters in *S. aureus* mediates drug resistance [41]. Thus, gene downregulation of this pathway may promote bacterial resistance.

Amino acid biosynthesis involves a series of enzymatic reactions that synthesize amino acids from other compounds. A slower rate of amino acid biosynthesis can reduce the generation of adaptive resistant bacteria [42,43]. The biosynthesis pathways of amino acids can also affect the metabolic status of other pathways such as the carbon metabolism pathway, and the reduced synthesis of related amino acids can decrease the tolerance of bacteria to antibacterial drugs [44]. Thus, downregulation of genes in this pathway may promote bacterial resistance. In this study, the differentially expressed genes in the amino acid biosynthesis pathway (i.e., the biosynthesis of valine, leucine, and isoleucine) were downregulated. This is consistent with Hua Xin’s study of the activity and mechanism of anti-MASR by transcriptome sequencing, where amino acid synthesis pathways (lysine, valine, leucine, and isoleucine), as well as genes related to *S. aureus* infection pathways, were significantly downregulated by prosopol [45], and the *ilv* operons encoding leucine, isoleucine, and valine intermediates were downregulated in *Escherichia coli* [46].

The citrate cycle, also known as the tricarboxylic acid cycle (TCA cycle), is a central metabolic pathway in bacteria that produces energy (ATP) and synthesizes biomacromolecular precursors such as 2-oxoglutarate [47]. The citrate cycle is associated with the virulence or toxicity of pathogens, in ways such as the production of major biofilm mucous substances [48] and polysaccharide intercellular adhesin (PIA). The loss of glutamate synthase prevents the formation of biofilms. Further studies have found that the loss of this enzyme causes iron deficiency due to the chelation of citrate ions in the citric acid cycle, thus inhibiting the formation of biofilms [49]. The mutation of the TCA circulating gene sucC increases the sensitivity of MASR to β-lactam antibiotics [50]. In this study, sucC gene expression was upregulated; the sucC gene regulates succinate coA ligase, thereby affecting the citric acid cycle pathway and leading to bacterial drug resistance. The expression levels of the sdhB and sdhA genes in the TCA cycle were upregulated, and sdhB and sdhA genes regulate the synthesis of succinate dehydrogenase. After the upregulated gene expression, the synthesis of related substances downstream may be accelerated; the citric acid cycle and energy metabolism will also be accelerated, and the production of adaptive drug-resistant bacteria will increase. Wang et al. [51] found that sdhB and sdhA were related to the formation of persistent bacteria in *S. aureus*. Studies have shown that the formation of persistent bacteria is an important cause of chronic infections, especially in *S. aureus* and *P. aeruginosa* [52]. The pdhA gene regulates the synthesis of pyruvate dehydrogenase that catalyzes the oxidative decarboxylation of pyruvate during the citric acid cycle [53]. The lipamide dehydrogenase (LPD) encoded by the lpdA gene is a component of the pyruvate dehydrogenase complex (PDHc), α-ketoglutarate dehydrogenase (AKGDH), and glycine cleavage multienzyme (GCV) systems [54]. In this study, the upregulated expression of pdhA and lpdA genes accelerated the synthesis of pyruvate dehydrogenase; this accelerated the citric acid cycle and thereby increased the production of drug-resistant bacteria.

Adaptive resistance works within bacteria by enabling rapid adaptation to non-lethal environmental stresses (such as concentrations of non-lethal antimicrobials), allowing the bacteria to survive longer and providing time and opportunity for the bacteria to develop specific and persistent resistance. Bacteria in the adaptive resistance state will produce different states of bacteria to resist the pressure of antibacterial drugs: tolerant bacteria, persistent bacteria, and resistant bacteria. Resistant bacteria have the ability to survive for a short time under high lethal doses of antibiotics [55]. In some studies, it has been shown that the emergence of resistant bacteria will further promote the emergence of drug-resistant bacteria, and resistant bacteria is considered to be one of the factors that produce drug-resistant bacteria [12]. Persistent bacteria refer to a small number of bacteria that survive under the action of high doses of antibiotics without genetic resistance [56]. Persistent bacteria are one of the main factors leading to chronic and recurrent infections in the body, and the persistence of bacteria is very likely to lead to the generation of drug-resistant bacteria [57]. The emergence of resistant bacteria and persistent bacteria makes the treatment of antibiotics more difficult. Therefore, exploring the molecular mechanism of adaptive resistance provides data support for controlling the generation of adaptive resistance.

## 5. Conclusions

In this study, three kinds of adaptive drug-resistant bacteria were screened using an in vitro drug dynamic model through different administration schemes, and an evolutionary process of adaptive resistance has been discovered: when the bacteria were subjected to antibiotic pressure, tolerant bacteria appeared first, followed by persistent bacteria and finally drug-resistant bacteria. Transcriptome sequencing showed that adaptive drug resistance involved a network regulatory mode in which multiple pathways worked together, and five network regulatory gene nodes were identified. The results provide guidance for clinical drug use in animals infected with *S. aureus*, and the findings can be used to explore adaptive drug resistance and the targets of network regulation.

## Figures and Tables

**Table 1:** Results of sensitivity test in vitro.

Name	Treatment (μg/mL)
MIC	0.5
MPC	1.6
MSW	0.5–1.6

## Data Availability

Data are contained within the article and Supplementary Materials.

## Supplementary Materials

The following supporting information can be downloaded at https://www.mdpi.com/article/10.3390/microorganisms13020329/s1, Table S1: Test results of bacterial RNA samples; Table S2: Significantly enriched differentially expressed genes in the KEGG pathway.
