# Supplementary material for: Adaptive Resistance of Staphylococcus aureus to Cefquinome Sulfate in an In Vitro Pharmacokinetic Model with Transcriptomic Insights
Source: Microorganisms. 2025 Feb 2;13(2):329. doi: 10.3390/microorganisms13020329 (PMC11858071; doi:10.3390/microorganisms13020329)
Supplement: Supplementary file 1 [file microorganisms-13-00329-s001.zip › microorganisms-3433409-supplementary.pdf]

Supplementary Material

1 Supplementary Tables

Table S1. Test results of bacterial RNA samples

Sample name	Concentration (ng/ul)	volume	A260/280	RQN value	result
To-1	100	63	1.82	7. 16	OK
To-2	100	69	1.94	6.94	OK
To-3	100	59	1.95	7.06	OK
RE-1	100	71	1.99	6.98	OK
RE-2	100	70	1.91	6.92	OK
RE-3	100	69	1.93	6.91	OK
PE-1	100	66	1.96	7.03	OK
PE-2	100	75	1.75	6.96	OK
PE-3	100	56	2.02	7.03	OK
OR-1	476.5	30	1.92	7.09	OK
OR-2	506.3	30	1.93	7.11	OK
OR-3	525.9	30	1.92	7.24	OK

Table S2. Significantly enriched differentially expressed genes in the KEGG pathway

Path name	Gene Name	Difference multiple	p-value
Ribosome			
A0A0E0VLH6	rplK	(+)12.614	＜0.05
A0A0E0VN72	rplL	(+)7.702	0.002
A0A0E0VQJ8	rplT	(+)6.646	0.001
A0A0E0VS56	rplQ	(+)10.876	0.000
A0A0E0VSJ0	rplN	(+)11.494	＜0.05
A0A0D1HS65	rplA	(+)9.109	＜0.05
A0A0E0VLB0	rpsR	(+)9.483	0.005
A0A0E0VPR0	rpsD	(+)8.240	＜0.05
A0A0E0VR82	rpsC	(+)11.141	0.000
Oxidative phosphorylation			
A0A0H3K7X9	qoxD	(+)9.375	0.000
A0A0E0VPI2	qoxC	(+)11.450	0.000
A0A0E0VPR6	sdhB	(+)9.596	＜0.05

A0A0E0VNL6	qoxB	(+)12.078	＜0.05
Citrate cycle (TCA cycle)			
A0A0E0VPR6	sdhB	(+)9.596	＜0.05
A0A0E0VQ49	sucC	(+)12.285	＜0.05
A0A0H2XHD5	pdhA	(+)12.196	0.001
A0A0H3JU98	lpdA	(+)12.826	＜0.05
A0A0E0VN68	sdhA	(+)10.673	0.000
Biosynthesis of amino acids			
A0A1Q4GZ82	argJ	(-)5.258	0.003
A0A0H2WWP5	B4602_RS06940	(-)3.454	0.004
A0A0H3JPZ3	B4602_RS06945	(-)2.755	0.002
A0A299MEY2	trpD	(-)2.967	0.005
A0A181EA30	trpB	(-)4.186	0.006
Q2FYP1	B4602_RS07075	(-)5.169	0.019
Q2FWK5	B4602_RS10770	(-)2.603	0.000

A0A0E0VSH0	ilvC	(-)4.343	0.009
X5EGZ0	B4602_RS10780	(-)5.449	0.007
A0A1M4RI36	ilvA	(-)4.941	0.010
A0A0H2XH65	rocF	(-)3.580	0.005
A0A0E0VSF9	B4602_RS12300	(-)3.839	0.000
ABC transporters			
A0A0H3JK74	sirB	(-)4.213	0.000
W8U7B3	phnC	(-)3.856	0.014
A0A0H2XK78	ugpC	(-)4.077	0.002
T1Y5P6	B4602_RS02150	(-)4.122	0.022
A0A0H3JSZ1	gmpC	(-)5.248	0.015
A0A0H3KBI1	B4602_RS03770	(-)3.061	0.002
A0A0H2XFV3	B4602_RS03775	(-)3.377	0.000
A0A0E1AJ06	B4602_RS03780	(-)3.013	0.005
T1Y8R3	B4602_RS05025	(-)5.021	0.002
A0A0H3KE05	B4602_RS10590	(-)3.543	0.002

A0A0H2XHX7	B4602_RS11480	(-)4.876	0.011
A0A0H2XJ16	B4602_RS13030	(-)5.196	0.003
A0A0H3JU11	B4602_RS14225	(-)4.172	＜0.05
A0A0H2XIR2	vraD	(-)4.550	0.000
Valine, leucine and isoleucine biosynthesis			
A0A0E0VSH0	ilvC	(-)4.343	0.009
X5EGZ0	B4602_RS10780	(-)5.449	0.007
Q2FWK5	B4602_RS10770	(-)2.603	0.000
A0A1M4RI36	ilvA	(-)4.941	0.010
Carbon metabolism			
A0A0H2XHW0	gap	(+)12.079	0.000
A0A0E0VNL1	B4602_RS04010	(+)12.005	＜0.05
A0A0H2XHD5	pdhA	(+)12.196	0.001
A0A0H3JU98	lpdA	(+)12.826	＜0.05
A0A0E0VN68	sdhA	(+)10.673	0.000

A0A0E0VPR6	sdhB	(+)9.520	0.000
A0A0E0VQ49	sucC	(+)12.268	0.000
A0A0H3KCX3	gcvPA	(+)9.306	＜0.05
A0A0E0VRY1	B4602_RS12730	(+)10.374	0.014
Carbon metabolism			
A0A0E0VMG9	B4602_RS01025	(+)11.867	0.000
A0A0H2XHW0	gap	(+)12.079	0.000
A0A0E0VNL1	B4602_RS04010	(+)12.005	＜0.05
A0A0E0VMG6	eno	(+)12.465	＜0.05
A0A0H2XHD5	pdhA	(+)12.196	0.001
A0A0H3JU98	lpdA	(+)12.826	＜0.05
A0A0H2XJ98	B4602_RS07230	(+)10.604	＜0.05
A0A0E0VRY1	B4602_RS12730	(+)10.374	0.014
